# Dataset of internal temperature during lithium-ion battery ageing

**DOI:** 10.1016/j.dib.2026.113403

**Published:** 2026-11-02

**Authors:** Begum Gulsoy, Timothy Vincent, Quang Ngo, Mona Faraji Niri, James Marco

**Affiliations:** WMG, University of Warwick, Coventry, CV4 7AL, United Kingdom

**Keywords:** In-situ monitoring, Core temperature, Advanced cell characterisation, Battery ageing

## Abstract

This paper presents an experimental dataset obtained from LG Chem INR21700 M50 lithium-ion cells subjected to ageing and performance characterisation tests. The dataset comprises six cells: three pristine cells and three instrumented cells with thermocouples embedded in the cell core. Ageing tests were conducted over a total of 550 cycles, comprising 100 cycles of short-term ageing and an additional 450 cycles of long-term ageing. Performance characterisation was conducted using a dynamic load profile derived from the operating conditions of a real vehicle in an urban environment over a 45-minute period. During both ageing and load-profile testing, key battery parameters, including cell voltage, current, discharge capacity at a 1 C discharge rate, cell surface temperature, and core temperature, were monitored. The dataset provides direct measurements of cell core temperature over 550 ageing cycles, enabling detailed investigation of the evolution of thermal characteristics with cell ageing. The combination of electrical, surface-temperature, and core-temperature measurements provides a comprehensive experimental dataset for investigating the coupled thermal and degradation behaviour of lithium-ion cells and supports the development and validation of battery thermal and degradation models.

Specifications Table

**Table:** 

Subject	Electrochemistry
Specific subject area	Characterization of lithium-ion batteries degradation by core temperature
Type of data	Raw data (.txt) and processed data (.mat)
Data collection	Ageing data were obtained through charge-discharge protocols conducted using a Digatron MCT cycler. Load-profile cycling data were collected using a dynamic load profile specifically derived for LG Chem INR21700-M50 cells, applied via a Bio-Logic VSP-300 cycler. All tests were performed in a climate chamber set to 25 °С. Prior to all tests, cells were allowed to reach thermal equilibrium over a period of 2 hours. Measurements include cell voltage, current, capacity, surface temperature and core temperature. All data is processed using MATLAB 2021b analytical software.
Data source location	Institution: Warwick Manufacturing Group (WMG), Energy Innovation Centre, University of Warwick City: Coventry Country: United Kingdom GPS coordinates for collected samples/data: 52.38363378953185, −1.5615186436655097
Data accessibility	Repository name: Mendeley Data Data identification number: 10.17632/bf6fksxpp2.2 Direct URL to data: https://data.mendeley.com/datasets/bf6fksxpp2/2
Related research article	Gulsoy, B., Vincent, T.A., Sansom, J.E.H. and Marco, J., 2022. In-situ temperature monitoring of a lithium-ion battery using an embedded thermocouple for smart battery applications. Journal of Energy Storage, 54, p.105260. https://doi.org/10.1016/j.est.2022.105260

## 1 Value of the Data

• This dataset provides direct measurements of lithium-ion battery core and surface temperatures over 550 ageing cycles, offering a novel insight into the evolution of internal thermal behaviour during long-term cycling. It enables a better understanding of cell thermal characteristics as degradation progresses.
• The dataset provides valuable experimental evidence for researchers, battery manufacturers, and engineers to investigate electrochemical–thermal behaviour during ageing. It can support the development and validation of thermal and degradation models, contributing to more accurate temperature predictions and improved battery thermal management strategies.
• The ageing results demonstrate that the proposed instrumentation method does not significantly affect cell degradation behaviour, supporting its suitability for long-term in-situ temperature monitoring. Additional testing under transient load conditions confirms the sensor's ability to capture dynamic thermal responses, strengthening confidence in the reliability of the measurements.

## 2 Background

The rapid development of the new energy sector has significantly increased global interest in lithium-ion batteries (LIBs), particularly for electric vehicles, aerospace, and grid storage. To meet the growing demand for higher energy density, researchers are advancing cathode and anode technologies, such as nickel- or manganese-rich cathodes and silicon-carbon anodes. While the material innovations increase energy capacity, they often compromise thermal stability, elevating the risk of safety issues such as thermal runaway [[1], [2], [3]].

In cylindrical lithium-ion batteries (LIBs), safety concerns are largely driven by thermal effects arising from heat generation within the cell. These thermal processes are closely coupled with electrochemical behaviour and can significantly affect battery performance, accelerate ageing, and contribute to various degradation mechanisms. For example, in high-rate charging and discharging scenarios, significant internal heat is generated, and due to the limited thermal conductivity of small cylindrical battery materials, core temperatures may rise significantly without being detected by surface-mounted sensors. The difference between surface and internal cell temperature makes it more difficult to assess the true thermal state of the cell, limiting the effectiveness of conventional monitoring approaches [3,4].

To address this challenge, our study presents a novel method for directly measuring core temperature using embedded sensors inside cylindrical cells, as detailed in [1]. This approach enables continuous monitoring of internal thermal behaviour throughout the cell ageing process. The dataset offers valuable insights into core temperature rise during the battery ageing process, supporting the refinement of thermal models, the evaluation of cooling system performance, and ultimately the enhancement of lithium-ion battery safety, reliability, and lifespan.

## 3 Data Description

The experimental dataset comprises three instrumented and three pristine LG-Chem INR21700-M50 cells. In this study, instrumented cells refer to the cells that were opened using a novel methodology, fitted with a bespoke K-type thermocouple (approximately 0.03°C resolution with operation range up to 1200°C), and resealed for characterisation testing, as further described in [1]. Pristine cells are unmodified with no sensors attached. Both pristine and instrumented cells are subject to identical cycling protocols: short-term and long-term ageing studies, with a reference performance test (RPT) conducted at intervals of 20 cycles and 50-cycles, respectively. However, load profile testing was performed only on the three instrumented cells.

The LG Chem INR21700-M50 is a cylindrical lithium-ion cell in the 21700-format, featuring a rated capacity of 5 Ah. It has a nominal voltage of 3.63 V and a mass of approximately 68.0 ± 1.0 grams. The cell has a charge cut-off voltage of 4.2 V and a discharge cut-off voltage of 2.5 V. Its operating temperature ranges are 0°C to +45°C for charging and −20°C to +60°C for discharging. The cell utilizes a lithium nickel manganese cobalt oxide (LiNiMnCoO₂) cathode, a graphite–silicon oxide (SiOx) composite anode, and a ceramic-coated separator for enhanced thermal stability and safety.

The file structure of the dataset is shown in Fig. 1. Within the parent folder *CoreTemperature_AgeingStudy*, there are three subfolders named *ShortTermAgeing_100cycles, LongTermAgeing_450cycles* and *LoadProfileTesting*. The subfolders for ageing studies contain cycle data and RPT data, which were conducted at 20-cycle and 50-cycle intervals, respectively. The *LoadProfileTesting* subfolder contains the current profile of a real vehicle operating in an urban environment, the derived current profile applied to the cells and the corresponding data table of test results.

**Fig. 1:**
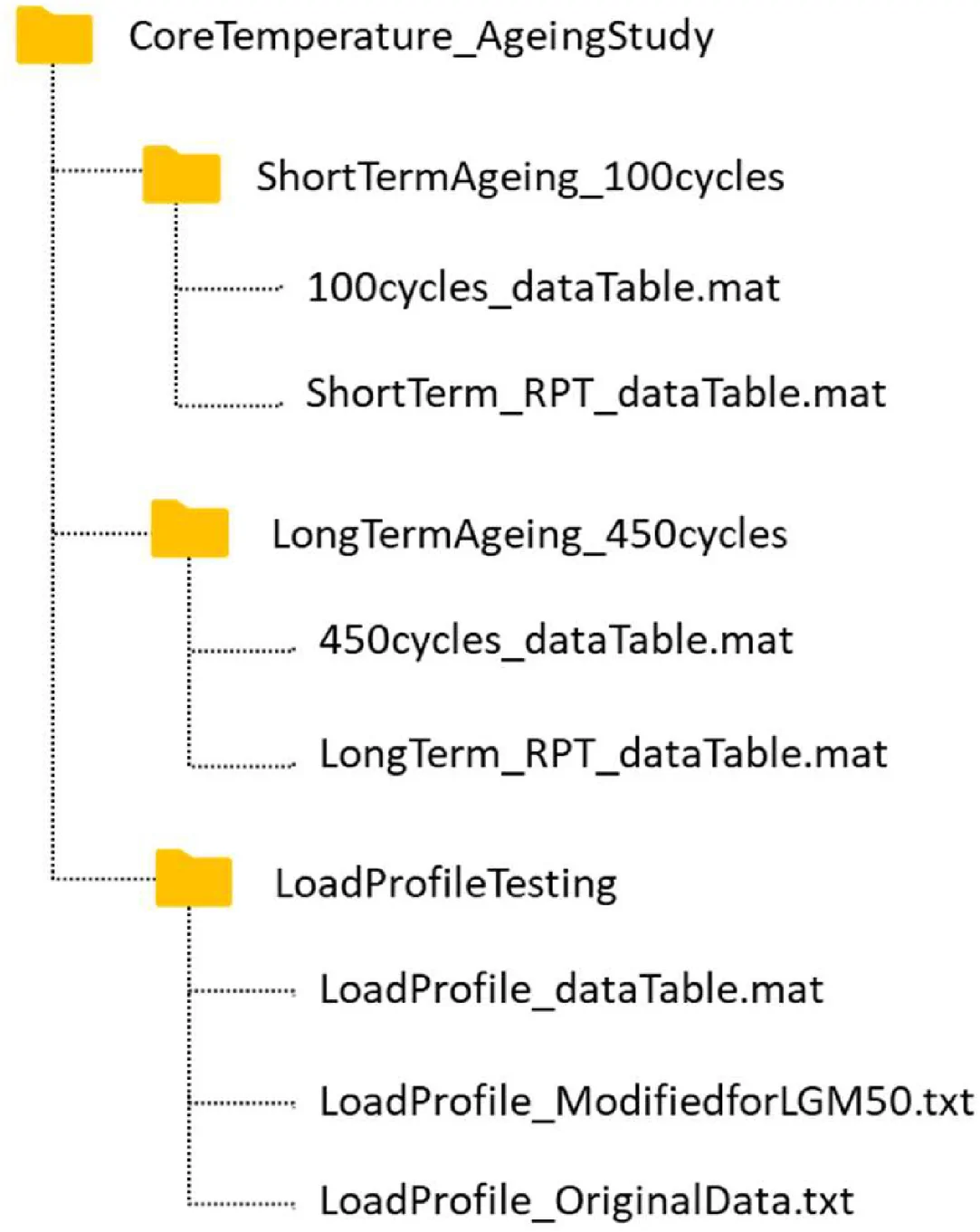
The file structure of the dataset.

The parameters across all datasets represent raw measurements collected directly from the cyclers and sensors via data loggers. Table 1 provides a detailed description of the parameters included in both the cycling and RPT datasets. The parameters for the ageing studies, from Step (column 6) to Core Temp (column 16), are presented as "*1 × x*" tables for each cell, corresponding to the experimental steps illustrated in Figure 2.

**Table 1:** Parameters in cycling and RPT datasets.

Column Number	Parameters	Unit	Details
1	Cell Name	−	ID assigned to each test cell
2	Condition	−	Indicates whether the cell is instrumented with internal sensors or non-instrumented cell
3	Sensor	−	Type of sensor used for instrumentation (e.g. thermocouple, pressure, thermistor, etc.)
4	Cell Manufacturer	−	Name of the company manufactured the cell
5	Type	−	Specific model of the cell
6	Step	−	Numerical identifier for experiment steps
7	Status	−	Descriptive label of experimental step (e.g. charging, discharging, rest, etc.)
8	Step Time	Seconds	Duration of experimental step measured in seconds
9	Prog Time	Seconds	Total elapsed time since the beginning of experiment
10	Cycle No	−	Count of completed charge/discharge cycles
11	Voltage [V]	Volt	Cell voltage measured during test
12	Current [A]	Amps	Applied current during test
13	Capacity [Ah]	Amp-hour	Measured cell capacity during test
14	Time Temp	hh:mm:ss	Time stamp corresponding to the recorded temperature values
15	Surf Temp [C]	°С	Surface temperature measured at the cell's midpoint (35 mm from the negative terminal)
16	Core Temp [C]	°С	Internal temperature measured at the cell's midpoint (35 mm from the negative terminal), inside the available cavity.

**Fig. 2:**
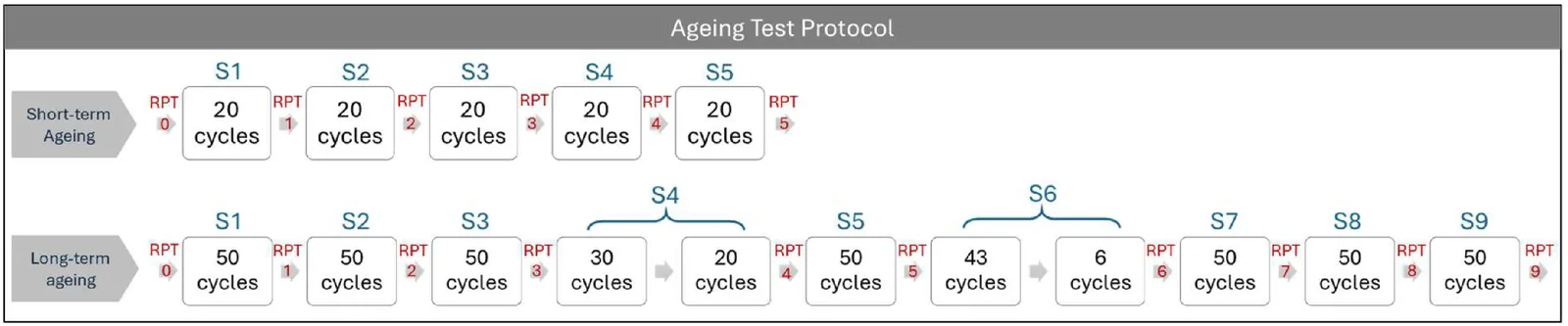
Ageing test protocol flowchart.

## 4 Experimental Design, Materials and Methods

### 4.1 Ageing tests

Ageing data were collected using charge-discharge protocols applied to LG-Chem INR21700-M50 cells. All tests were performed in a thermal chamber with the ambient temperature controlled to 25°C. A total of six cells were subjected to short-term ageing over 100 cycles, including three cells instrumented with internally located thermocouples and three pristine cells. During cell cycling, charge and discharge rates of C/3 and 1C were employed respectively. All cells were charged to the upper voltage limits of 4.2 V using a CC-CV procedure. Termination of the CV stage occurred when the applied current reduces below C/20. During discharge, a CC current was applied to the cells until a lower cut-off voltage of 2.5 V was reached. A rest period of 1 min was allowed before commencing the next charge-discharge cycle. At intervals of 20 cycles, the standardised RPT protocol, as detailed in [5], was conducted to evaluate the cell’s retained capacity and direct current internal resistance (DCIR). The ageing investigation was extended by an additional 450 cycles as part of the long-term ageing study. All cells were cycled under the same conditions. However, RPT tests were conducted every 50 cycles. Fig. 2 shows the ageing test protocol. Experiments S4 and S6 were terminated before reaching the intended number of cycles due to unknown interruptions. However, no measurement data are missing from the recorded cycling periods. To complete the planned 50-cycle dataset, additional cycles were subsequently performed and recorded. Consequently, all available data are included in the dataset, with only the S4 and S6 data provided in separate files to distinguish the continuation test from the original cycling sequence.

All tests were performed in a climate chamber set to 25 °С. Prior to all tests, cells were allowed to reach thermal equilibrium over a period of 2 hours. Measurements include cell voltage, current and capacity were recorded using a Digatron MCT cycler, whereas surface temperature and core temperature were recorded by Digatron DLT. All parameters were logged at the frequency of 1 Hz. Cell surface temperature was monitored using K-type thermocouples (363-0250, RS Pro) attached at the centre of the cell surface, while core temperature was measured using calibrated bespoke embedded K-type thermocouples (approximately 0.03°C resolution with operation range up to 1200°C).

All bespoke thermocouples were calibrated over the temperature range of 20 and 50°C, covering the nominal operating range of the cells. Calibration was carried out in a climate chamber (Binder MKF56). For accurate temperature reference, a K-type thermocouple (±0.5°C accuracy up to 50°C) was co-located with each research thermocouple. The chamber temperature was increased in 5°C increments at 120 min intervals. The results presented in [1] show good correlation between the bespoke thermocouples and the commercial reference sensor. The overall measurement uncertainty was determined to be ±0.143°C and is included in the reported sensor accuracy.

#### 4.1.1 Load profile tests

A dynamic load profile was derived from the data reported in [6], which represents a current profile of a real vehicle operating in an urban environment over 45 minutes. The current measurements were extracted from the profile and subsequently scaled (from vehicle-level current data to the single-cell level) to suit the maximum charge and discharge currents for LG-M50 cell by the manufacturer. This was achieved by multiplying the original current values by a factor of 0.08282. The resulting modified load profile comprised transient current of up to 2.5 A during charging and 7.5 A during discharging. Further experimental details are fully described in [1], they are not reproduced in this manuscript. This modified profile was applied at various SOC threshold, specifically 100 %, 80 % and 50 %. The load-profile was applied to the cells using an Urban Profile function within the Modulo Bat technique, implemented via a Bio Logic VSP-300 cycler.

All tests were conducted in a climate chamber maintained at 25°C, with a 2-hour equilibration period prior to testing. The measurement of cell voltage, current, and capacity was recorded at a frequency of 1 kHz during the application of the load profile. Data acquisition during the discharging stages for SOC adjustment steps was conducted at a frequency of 1 Hz. Cell surface and internal temperatures were measured using a PicoLogger TC-08 at a sampling frequency of 10 Hz.

## Limitations

The study was conducted using a total of six cells, comprising three pristine cells and three instrumented cells with thermocouples embedded in the cell core. The limited sample size restricts the assessment of cell-to-cell variability and statistical reproducibility, although consistent capacity-fade trends were observed within each group. Despite this limitation, the dataset has attracted interest from modelling researchers for model development and validation. Therefore, while the dataset provides valuable experimental measurements, the small sample size should be considered when interpreting the results and assessing their broader applicability.

## Ethics Statement

The proposed data does not involve any human subjects, animal experiments, or data collected from social media platforms. The authors confirm that this work meets the ethical requirements of the journal*.*

## CRediT Author Statement

**B. Gulsoy:** Conceptualization, Methodology, Formal analysis, Investigation, Writing - Original Draft. **T.A. Vincent:** Writing - Review & Editing, Investigation. **Q. Ngo:** Data Processing. **M.F. Niri:** Data Processing, Writing - Review & Editing. **J. Marco:** Funding acquisition, Supervision, Resources, Writing - Review & Editing.

## Data Availability

Mendeley DataDataset of internal temperature during lithium-ion battery ageing (Original data).

## References

[1] Gulsoy B., Vincent T.A., Sansom J.E.H, Marco J. (2022). In-situ temperature monitoring of a lithium-ion battery using an embedded thermocouple for smart battery applications. J. Energy Storage.

[2] Gulsoy B., Chen H., Briggs C., Vincent T.A., Sansom J.E., Marco J. (2024). Real-time simultaneous monitoring of internal temperature and gas pressure in cylindrical cells during thermal runaway. J. Power Sourc..

[3] Gulsoy B., Vincent T.A., Briggs C., Sansom J.E., Marco J. (2023). In-situ measurement of internal gas pressure within cylindrical lithium-ion cells. J. Power Sourc..

[4] Xiao Z., Liu C., Zhao T., Kuang Y., Yin B., Yuan R., Song L. (2023). Online monitoring of internal temperature in lithium-ion batteries. J. Electrochem. Soc..

[5] Gulsoy B., Vincent T., Briggs C., Kalathingal A., Niri M.F., Marco J. (2025). Dataset of accumulated internal gas pressure and temperature during lithium-ion battery operation and ageing. Data Br..

[6] Niri M.F., Bui T.M., Dinh T.Q., Hosseinzadeh E., Yu T.F., Marco J. (2020). Remaining energy estimation for lithium-ion batteries via Gaussian mixture and Markov models for future load prediction. J. Energy Storage.

